# Editorial: Imagination, cognition, and the arts

**DOI:** 10.3389/fnhum.2024.1523760

**Published:** 2024-11-26

**Authors:** Víctor Bermúdez, Renata Gambino, Benito García-Valero, Grazia Pulvirenti

**Affiliations:** ^1^Department of Spanish Language, Section of Literary Theory and Comparative Literature, University of Salamanca, Salamanca, Spain; ^2^Department of Humanities, DISUM, Section of German Studies, University of Catania, Catania, Italy; ^3^Department of Spanish Language, General Linguistics and Theory of Literature, University of Alicante, Alicante, Valencian Community, Spain; ^4^University of Catania, Catania, Italy

**Keywords:** imagination, cognition, arts, literature, imaging

Imagination has long been and continues to be an enigmatic concept, frequently addressed but rarely fully elucidated, even by prominent philosophers such as Aristotle, Hume, Kant, and Husserl. Its definition and interpretation changes significantly across different heuristic frameworks—philosophical, aesthetic, poetic, and cognitive. The diverse discourses on imagination have generated persistent, unresolved questions regarding its modalities and effects, particularly concerning the construction of aesthetic and symbolic experiences. Symbolic experiences emerge from our capacity to manipulate tangible elements and perceptions of the real world, exploring through imagination their latent potentialities. During the 1970s, imagination was often undervalued, as noted by Huppauf and Wulf:

In the aftermath of the radical sixties, skepticism toward the social and political power of the imagination became popular. A political interpretation of the imagination created great expectations but they, it turned out, were mistaken... The imagination has had an uneven and controversial history in the modern period. For most of the time, it was considered secondary and ancillary and sometimes even a dangerous human faculty... It was placed among the weaknesses of the human nature and, at the same time, seen as an origin of the fear of losing control over reality. It seemed irreconcilable with the idea of self-determination through the production of knowledge in scientific disciplines (see Huppauf and Wulf, 2009, p. 1–2).

Conversely, in recent decades we have witnessed sustained investigation into imagination within cognitive studies, evolutionary anthropology, and cognitive and developmental psychology. Imagination has mainly been recognized as a core representational activity of the mindbrain. Numerous hypotheses and theories underscore different characteristics and functions of imagination (for review, see Brann, 2017; Kearney, 1988; Stevenson, 2003). Some scholars posit that imagination mediates the relationship between perceptions and mental representations, contributing to the construction of the “human imagination spectrum” (McGinn, 2004) and the “multidimensional continuum view” (Nigel, 2014). These frameworks suggest that imagination plays a crucial role in forming a stable representation of the world for each perceiving individual. Imagination, is therefore intended as the cognitive ability which empowers humans to creatively shape their experiences, perceptions, and visions of self and world, as well as memories and emotions.

Given its fundamental role in human existence, imagination has gradually become a focal point in contemporary cognitive research. Scholars like Mark Turner emphasize the epistemic necessity of investigating imaginative processes to not only unravel this profound enigma of the human mind brain but also to elucidate other cognitive faculties that depend on imagination (Turner, 2014). In its vast array of activities, imagination is understood as a complex emergent cognitive faculty intricately linked with perception, memory, and consciousness. It is considered as shaping human life by expanding the boundaries of our perceptual, affective understanding, influencing our actions, memories and our very being. In accordance with the thesis of Lakoff and Johnson, imagination functions by shaping “categorization, reason, propositional and non-propositional forms of thought,” through the employment of metaphor and narrative (Lakoff and Johnson, 1980, p. 193).

By manipulating and transforming perceptions, experiences and elements of the real world into counterfactual images, imagination reutilizes the somatosensory and motor systems (Gosetti-Ferencei, 2018; Gallese, 2019, p. 113–127). This imaginative manipulation involves the embodied activation of the sensorimotor system, which is reutilized when the mind processes counterfactual images, elaborates them through language and various media, or engages with mental imagery (Kuzmičová, 2014, p. 275–293) elicited by literature, visual artworks and different media.

Imagination can be observed and described as a complex system exhibiting features known as “self-organization” or “emergence,” involving the activation of multiple neural circuits engaged in higher associative cognitive functions. These fundamentally chaotic or complex systems have the capacity to produce patterns that appear non-chaotic and predictable. Drawing on the work of Varela and Thompson, every living organism is an enactive and imagining being, integrating a series of similar processes. From this perspective, imagination can be considered an integrated and dynamic flow of sensorimotor, memorial, visual, and eidetic activations (Varela et al., 1991; Dennett and Marcel, 1992; Pöppel and Kerstin, 1995; Varela and Depraz, 2003):

Mental acts are characterized by the concurrent participation of several functionally distinct and topographically distributed regions of the brain and their sensorimotor embodiment. […] It follows that one cannot hope to find a naturalized account of imagination as some sort of cognitive module or brain region. It must necessarily correspond, instead, to a dynamic, emerging global pattern that is able both to integrate the body/brain activity at a large scale and subside rapidly, for the benefit of the next moment of mental life. (Varela and Depraz, 2003, p. 201–202).

This means that the function of imagination, in a simplified form, is to shape the vast and diffuse experience of reality into a coherent mental-cognitive state. This process involves the formation of an assembly that integrates or discards other concurrent neural activities generated either exogenously or endogenously (Thompson, 2007, p. 79).

Through this dynamic, inferential, and integrated process of imagination, the subject constructs the imaginative figuration of the world and their existence within it as an autopoietic being (Maturana and Varela, 1980). Imagination, akin to perception, memory, consciousness, and cognition, along with other related enactive functions and processes, contributes to creating reproductive consciousness and the multidimensional spectrum of the world. Moreover, imagination, defined as the cognitive capacity to generate images, ideas, and sensations in the absence of direct sensory input, constitutes a fundamental pillar of human creativity and innovation. This faculty enables individuals to surpass the constraints of the experience, envisioning possibilities that extend beyond the tangible and immediate.

Among the manifold modes of imagination, we consider it to be an emergent and embodied process significant to any human experience of reflecting on and transforming the world, whether in thinking or in making artifacts. Imagination turns out to be intertwined with the embodied life “through the examples of explicitly embodied imagining in performance art, dance, and the making of film, as well as the evocations of embodiment through painting, literature, and social responsiveness” (Gosetti-Ferencei, 2018, p. 23–24). It plays a significant role not only in “representing” to the “mind's eye” what exists in reality, but also what does not exist and is evoked out of the sphere of pure potentialities of the “invisible” (Franzini, 2001).

From this perspective, human imagination has the capacity to model the invisible by creating *eidola*. The indistinct horizon of unexpressed potentiality in the realm of the invisible is directly related to the visible, encompassing the hidden aspects of a perceived object. The image, as a whole, emerges out of the imaginative processes containing elements that are not directly visible. These premises enable the analysis of images by tracing the process of their emergence within a horizon of meaning that, while exceeding empirical observation, finds fulfillment in sensory experience. From this perspective, the invisible is not the unknown or unspeakable but rather a pre-categorical dimension that serves as the source of all acts of imagination:

Imagination is one of the quintessential qualities of life and of our being. Its central attribute is the manifestation of vivid, lived mental content that does not refer directly to a perceived world but to an absence that it evokes. (Varela and Depraz, 2003, 195)

In this perspective, imagination plays a central role in the construction and perception of aesthetic objects and literary texts. Literary imagination is not only relevant for literary scholars. Many philosophers (Engell, 2014) and neuroscientists (Abraham, 2020) have found in literary imagination a study object and developed both conceptual frameworks and empirical methodologies to approach this complex human faculty at different levels of specificity and based on its relationship with other cognitive processes like perception, memory, emotions or consciousness itself. Artistic and literary imagination unfolds the most varied materials such as dreams, hallucinations, atmospheres, the incomplete, the invisible, fictional realms, former realities, etc., and in doing so literature, and the arts underline the human capacity to elaborate images of both existing and non-exciting entities, or to conceive hypothetical scenarios, actions or events that expand our consciousness. In this respect, literary and artistic imagination, involving different media, such as films, theatrical performance, video games, etc., fosters the development of a wide range of cognitive faculties that prove to be relevant for human adaptation and performance in the external reality.

Because of its extensiveness, the topic of imagination requires a transdisciplinary approach, joining neuroscientific and cognitive research with studies on aesthetics, philosophy, and the fine arts. In fact, the ambiguity and abundance of meanings comprised in artistic works strongly engage the imagination of the image beholder and of the reader, triggering their cognitive faculties and embodied simulation. The inquiry into imagination needs the contribution of scholars in both the scientific and the humanities fields, acknowledging conceptual and experimental approaches through several epistemological perspectives. This Research Topic of *Frontiers in Human neuroscience*, and specifically in *Cognitive Neuroscience* was meant to address this complex question, opening to contribution in both human and scientific research fields. It gathers six articles facing the topic from different perspectives, but all revealing that the research about imagination links body, brain, existing and non-existing realities into a unique experiential bundle, deeply influencing our everyday behaviors and world construction.

Kakimova and Salgaro's exploration faces the question of how counterfactual historical fiction affects cognitive processing. The two researchers focus on the perception of historical realism and the evocation of aesthetic emotions such as surprise. Through eye-tracking experiments, the study investigates how readers reconcile real and imagined worlds on Robert Harris's novel *Fatherland*. The findings reveal that counterfactual fiction, by contrasting with known historical facts, triggers more cognitive effort and self-reflection than conventional historical narratives. It also suggests a reduction in the readers' receptivity to fascist ideology and superstitions, enhancing critical thinking processes in a critical time such as our decade, where extremist political discourses are gaining ground.

Martínez, in the only solo paper of this Research Topic, presents an investigation on how the physiological descriptions of characters' emotions in literary narratives activate readers' mental imagery and emotions. By analyzing two fictional texts, *Harry* and *Atonement*, the study highlights the metonymic connection between physiological symptoms (like trembling) and emotion schemata (like fear), emphasizing how these descriptions enhance readers' embodied experience of the narrative. In connection with the latest premises formulated in the field of neurohermeneutics, her findings suggest that such physiological cues increase opportunities for emotional engagement, as readers draw from personal experiences to imagine and feel the emotions depicted.

The value of theatrical practice on interpersonal synchronization behaviors in actors and non-actors is investigated by Sofia et al.. They put into test how acting experience enhances the ability to establish effective relationships, manage personal and others' emotions, and maintain a sense of presence during interactions. Using a joint task called Random Sequence Generation (RSG), the research reveals that actors perform better in managing feelings of entitativity and presence. The results suggest that theatrical training improves social skills and emotional regulation, benefiting both artistic and everyday interactions, which provides new ground for utility of theater in human understanding in the age of distant connections among humans, enabled by today's technology.

The anticipatory thinking that literature and the arts elicit has a long tradition in philosophy, and literary studies. The hermeneutical circle, for instance, as Schleiermacher, Dilthey, or Gadamer conceived it, describe the interpretation of cultural materials as a constant and renewed anticipation and reconstruction process (Zimmermann, 2015). Such anticipatory thinking is at the core of Bortolotti et al.'s contribution “*Imagination vs. routines: festive time, weekly time, and the predictive brain*.” Here, the authors elaborate a conceptual framework which relates social, artistic and cultural practices with neurobiological functions of prediction and social cognition. The study argues that traditions and social conventions play a substantial role in anticipation both as a practice and as a cognitive process, which carries evolutionary value for humans, and it opens a line for further research on neuroimaging of shared predictions. The paper proves evidence on how art facilitate learning, play, and simulation faculties and how predictive cultures provide coherence and cohesion to society.

The brain's capacity to identify the self and relate it with the others is at the basis of human understanding. In this line, Portillo's contribution approaches imagination through the cognitive processes of empathy in the context of film studies to argue that “film fictions are particularly effective at eliciting emotions” (2). Her discussion examines the advantages and constrains of three models for the study of cinematic empathy, namely embodied simulation, resonance-enactment, and cinema of empathy. Portillo concludes that empathy plays a substantial role not only on the engagement with film fictions but also that cinematic imagination creates bounds with the external social realities.

Ambiguity proves to be a formal feature in artistic production that highly stimulates the brain as it opens a space for interpretation and speculation. How to deal with the indeterminate is one of the cognitive functions involved in literary imagination. The paper by Zheng “*Prototype theory and the importance of literary form for moral imagination*” investigates fuzzy conceptual boundaries arguing for a prototype theory for moral imagination in order to understand how we make sense of literary ambiguities. The paper relates literature with moral philosophy and discusses literary examples in which particularities and universals provide a frame to decode ambiguity by means of prototypes and how this is relevant at a cognitive level.

To conclude, in all papers, a central focus is represented by the connections relating social, artistic and cultural practices with imagination and embodied processes. This argument is especially relevant today, when the debate of Artificial Intelligence is pervading almost every aspect of science and social life. The enactive approach to cognition and imagination is incompatible with the belief that machines can engage in imaginative activity analogously to living beings. From the theoretical perspective we are assuming in this paper, imagination cannot be divorced from perception, memory, emotions and sensations: neither does cognition nor intelligence. In other words, it is not possible to imagine intelligence without a *heart*, so the term of “artificial intelligence” becomes problematic as the word “intelligence” is used in the fashion of the first cognitivist era, dominated by the behavorist paradigm, before embodied approaches to cognition came to the foreground of biological sciences. Therefore, it can be said that imagination, together with the production of natural language, is the frontier of artificial intelligence for the challenges that this discipline and technology is facing in the present.
